# Cytoplasmic and Nuclear Localization of TCTP in Normal and Cancer Cells

**DOI:** 10.1155/2012/871728

**Published:** 2012-05-15

**Authors:** Yu-Ping Ma, Wu-Ling Zhu

**Affiliations:** Department of Pathology, Xinxiang Medical University, Henan 453003, China

## Abstract

*Objective*. Intracellular localization of translationally controlled tumour protein (TCTP) was investigated in cancer cells. *Methods*. The expression and localization of TCTP were detected at 12 h, 24 h, 48 h, 60 h time points in culture of human hepatocarcinoma cell line HepG2, human cervical carcinoma cell line HeLa, and human normal liver cell line HL-7702 by immunofluorescence. *Results*. TCTP was expressed in both normal and tumor cells, and its localization changes at different time points. TCTP was mainly expressed in cytoplasm from 24 h to 48 h then expressed in both nucleus and cytoplasm at 60 h in HL-7702 cells. While in HepG2 cells, TCTP first localized at cell membrane within 24 h then at both nucleus and cytoplasm from 48 h to 60 h; TCTP localized at both nucleus and cytoplasm from 12 h to 60 h in Hela cells. *Conclusion*. The translocation of intracellular expression of TCTP in normal and tumor cells at different time points may pave a path to the studying of TCTP role in tumor growth.

## 1. Background

The translationally controlled tumor protein (TCTP), a highly conversed protein [1, 2], also called the histamine releasing factor (HRF) [3], has been suggested as a tumor-associated antigen and widely expressed in mammals as well as in a wide range of other organisms of both animal and plant kingdom [4]. Its mRNA and protein expression levels tend to be higher in the colorectal cancers (CRCs) [5] and hepatocellular carcinoma [6], compared to the corresponding normal tissues. TCTP was found to be the most strikingly downregulated in tumor reversion [7]. Moreover, the level of TCTP in the revertants from three other major solid cancers, colon, lung, and melanoma cell lines, has the same results [5, 8, 9]. In addition, transfection with antisence of TCTP attenuated malignancy of v-src-transformed NIH3T3 cells. Recently, TCTP has attracted the attention of an increasing number of researchers interested in various biologically and medically relevant processes. This is largely due to the fact that TCTP levels are highly upregulated in response to a wide range of extracellular stimuli [10, 11]. A series of recent reports highlighted the importance of TCTP for cell cycle progression and malignant transformation [12, 13]. TCTP was also shown to display an extracellular function as a histamine release factor and to have antiapoptotic activity [14].

 The intracellular localization of TCTP remains controversy. It was shown to localize in cytoplasm by Arcuri et al. [15] and nucleus by Li et al. [14], respectively. TCTP can be secreted and tumor-suppressor-activated pathway-6 (TSAP6) facilitates the secretion of TCTP via a nonclassical pathway through exosomes which highlights the association of TCTP and TSAP6 in cytoplasm [16]. The purpose of our study was to determine the localization of TCTP expression in two human cancer cell lines and one normal cell line. We applied cell immunofluorescence and detected the protein expression of TCTP at different time points in these three cell lines. We found that TCTP localized in both cytoplasm and nucleus and its translocation varied between normal and tumor cell lines at different time points.

## 2. Materials and Methods

### 2.1. Cell Culture

 Human hepatocellular carcinoma cell line HepG2, human cervical cancer cell line HELA, and human normal hepatocyte cell line HL-7702 were purchased from Chinese Academy of Science (Shanghai) and grown in Dulbecco's Modified Eagle Medium (DMEM) (GIBCO, Invitrogen, USA) supplemented with 10% FBS (ExCell, Genetimes, China), maintained at 37°C, 95% humidity, and 5% carbon dioxide.

### 2.2. Reagent and Antibodies

 The rabbit polyclonal antibody for TCTP, rabbit polyclonal antibody for TSAP6, and goat polyclonal secondary antibody to rabbit IgG-H & L (FITC) were purchased from Abcam. Paraformaldehyde and Tween20 were purchased from Sigma-Aldrich. The 4,6-Diamidine-2-phenylindole dihydrochloride (DAPI), Bovine Serum Albumin (BSA), antifade mounting medium, and phosphate-buffered saline were purchased from Roche, Solarbio, Beyotime, and Zhongshan Golden Bridge Biotech, respectively.

### 2.3. Immunostaining and Microscopy

For indirect immunofluorescence, HepG2, HL-7702, and HeLa cells were cultured on coverslips and fixed with 4% paraformaldehyde after 12 h, 24 h, 48 h, and 60 h culture, respectively, then permeabilized with 0.2% Tween 20, blocked with 1% BSA for 1 h, and incubated with the rabbit polyclonal to TCTP (1 : 500) and TSAP6 (1 : 200), respectively, overnight at 4°C, coupled with the secondary antibody at 4°C for 6 hours. DAPI was used to stain the nucleus. Immunofluorescence staining imaging was captured using Laser Confocal Scanning Microscope (LCSM).

## 3. Results

### 3.1. Localization of TCTP

**Figure 1:**
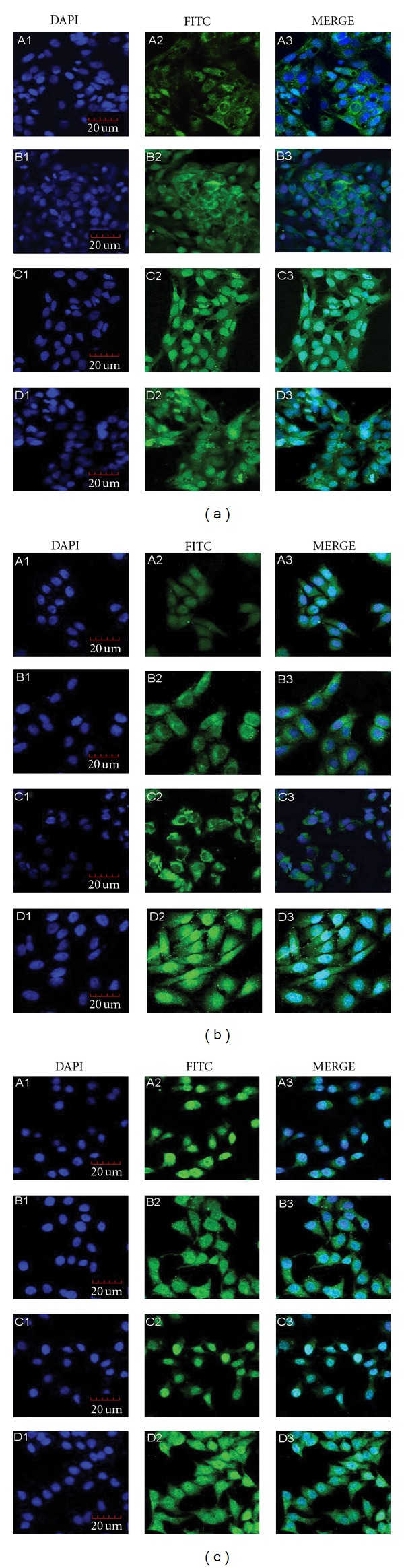
Localization of TCTP in HepG2 cells (a), HL-7702 cells (b) and HeLa cells (c), A1–A3: 12 h, ×400; B1–B3: 24 h, ×400; C1–C3: 48 h, ×400; D1–D3: 60 h, ×400.

As shown in Figure 1, TCTP protein was mainly localized in the cytoplasm and membrane from 12 h to 24 h, then in both nucleus and cytoplasm from 48 to 60 h in HepG2 cells (Figure 1(a)). In HL-7702 cells, the localization of TCTP protein showed a “nucleus-cytoplasm-nucleus” pattern at different time points (Figure 1(b)). TCTP localized in nucleus and cytoplasm in HeLa cells at every time point (Figure 1(c)).

## 4. Discussion and Conclusion

Previous reports from Arcuri's group [15] have demonstrated localization of TCTP protein in human prostate and prostate cancer cells using immunohistochemistry and immunofluorescence staining. The protein was mainly expressed in the secretory luminal epithelial and basal layer cells. A significant amount of protein was present in the prostatic fluids. Subcellular distribution studies on prostate epithelial cells showed the protein localized in the cytoplasm in interphase and colocalized with tubulin during mitosis. Li's group [14] has demonstrated that the intracellular localization of TCTP was present predominantly in the nucleus in HeLa cells after transfection with exogenous TCTP expression plasmid. Amson et al. and Ohgami et al. found that TCTP was secreted through an endoplasmic reticulum/Golgi-independent or nonclassical pathway and that the secreted TCTP was originated from preexisting pools. TSAP6, a p53-inducible 5-6 transmembrane protein [17, 18], was found to interact with TCTP and they partially colocalized in exosomes, some vesicular-like structures at the plasma membrane and around the nucleus. Functionally, the overexpression of TSAP6 consistently leads to enhanced secretion of both endogenously and exogenously expressed TCTP [16, 17]. We initiated our work by asking whether TCTP expression can be translocated and what is the relationship between its secretion level and function. For the first time, using the approach of immunofluorescence staining, we found that TCTP localized at different part within cells via several experiments. As demonstrated above, TCTP protein expression could be translocated along with cell growth.

 Interesting phenomena of our results were that TCTP localization differed between cell lines at the same time points and that TCTP did not change its cellular localization in HeLa cells, which can be explained as follows. (1) Different characteristics of cell lines. Firstly, HeLa cell is immortal because of being infected by the human papilloma virus (HPV). Unlike HeLa, HepG2 cells and HL-7702 cells have different cell cycles and are easy to die. Secondly, different cell lines have different cell cycles in which TCTP plays a key role. (2) Different TCTP expression levels. TCTP is an antiapoptotic and conserved protein. Numerous studies have shown that TCTP expression level in tumor is higher than that in the corresponding normal tissues, and inhibition of TCTP expression can attenuate malignant phenotypes [7], indicating TCTP has a critical role in tumorigenesis. Therefore, translocation of subcellular TCTP at different times might reflect its biological functions, and the underlying mechanism needs further investigation.

 In summary, we reported the localization and dynamic translocation of TCTP in different cell lines. TCTP localized in both cytoplasm and nucleus and it translocated into different subcellular units along with cells growth. Our finding provides more evidence showing the association between TCTP localization and function, improving our understanding of the important role of TCTP in cancer formation.

##  Authors' Contribution

Y.-P. Ma carried out the experiments and cowrote the paper; W.-L. Zhu directed the research and cowrote the paper. All authors read and approved the final paper.
